# In Vitro Insights into the Anti-Biofilm Potential of *Salmonella* Infantis Phages

**DOI:** 10.3390/antibiotics14080744

**Published:** 2025-07-24

**Authors:** Jan Torres-Boncompte, María Sanz-Zapata, Josep Garcia-Llorens, José M. Soriano, Pablo Catalá-Gregori, Sandra Sevilla-Navarro

**Affiliations:** 1 Centro de Calidad Avícola y Alimentación Animal de la Comunidad Valenciana (CECAV), 12539 Castellón, Spain; j.torres@cecav.org (J.T.-B.); m.sanz@cecav.es (M.S.-Z.); j.garcia@cecav.org (J.G.-L.); p.catala@cecav.org (P.C.-G.); 2 Food & Health Lab, Institute of Materials Science, University of Valencia, 46980 Valencia, Spain; jose.soriano@uv.es; 3 Joint Research Unit on Endocrinology, Nutrition and Clinical Dietetics, University of Valencia-Health Research Institute La Fe, 46026 Valencia, Spain; 4 Departamento de Producción y Sanidad Animal, Salud Pública Veterinaria y Ciencia y Tecnología de los Alimentos, Instituto de Ciencias Biomédicas, Facultad de Veterinaria, Universidad Cardenal Herrera-CEU, CEU Universities, 46113 Moncada, Spain

**Keywords:** biofilms, *Salmonella*, infantis, bacteriophages, phages, food safety

## Abstract

**Background/Objectives**: As bacteriophage-based strategies to control bacterial pathogens continue to gain momentum, phage therapy is increasingly being explored across various fields. In the poultry industry, efforts to minimize the public health impact of *Salmonella* have spurred growing interest in phage applications, particularly as prophylactic and disinfecting agents. Although the disinfecting potential of bacteriophages has been recognized, in-depth studies examining their efficacy under varying environmental conditions remain limited. This study focused on evaluating the effectiveness of bacteriophages as disinfecting agents against biofilm-forming *Salmonella* Infantis under different environments. **Methods**: A comprehensive screening of biofilm-producing strains was conducted using Congo Red Agar and 96-well plate assays. Two strains with distinct biofilm-forming capacities were selected for further analysis under different environmental conditions: aerobic and microaerobic atmospheres at both 25 °C and 37 °C. The resulting biofilms were then treated with four phage preparations: three individual phages and one phage cocktail. Biofilm reduction was assessed by measuring optical density and CFU/well. Additionally, scanning electron microscopy was used to visualize both untreated and phage-treated biofilms. **Results**: The results demonstrated that all *S*. Infantis strains were capable of forming biofilms (21/21). All three phage candidates exhibited biofilm-disrupting activity and were able to lyse biofilm-embedded *Salmonella* cells. Notably, the lytic efficacy of the phages varied depending on environmental conditions, highlighting the importance of thorough phage characterization prior to application. **Conclusions**: These findings underscore that the effectiveness of bacteriophages as surface disinfectants can be significantly compromised if inappropriate phages are used, especially in the presence of biofilms.

## 1. Introduction

Salmonellosis is one of the most relevant foodborne illnesses worldwide; thus, it is a major cause of significant socio-economic losses and a threat to human health, livelihood, and health care systems [1,2]. The disease is caused by *Salmonella* spp., a gram-negative enterobacteria ubiquitous in domestic animals that can survive under a wide range of physical conditions [3,4]. *Salmonella* infections in humans are predominantly associated with the consumption of animal-food products, and although it is present in all main livestock production, poultry and its derived products are frequently linked to salmonellosis cases in humans as sources of infection [1,2,5].

There are over 2500 known *Salmonella* serovars; however, serovars Enteritidis, Typhimurium, monophasic Typhimurium, and Infantis are highly relevant for public health due to their high prevalences in human cases [6,7]. According to the European Food Safety Authority (EFSA), *Salmonella* Infantis was the fourth serovar most frequently involved in human salmonellosis in 2023. It caused 682 (2.0%) European Union-acquired cases and was strongly associated with broilers at primary production [5]. The worldwide detection and isolation trends of serovar Infantis have been increasing since 2011 due to the plasmid of emerging *Salmonella* Infantis (pESI). The megaplasmid carries antimicrobial resistance (AMR) genes, such as extended-spectrum beta-lactamase coding genes (ESBL), and virulence factors like heavy metal tolerance, that enhance bacterial survival and pathogenicity. Moreover, due to the selective pressure of *S*. Infantis in poultry farming, a new version of the plasmid called pESI-like, containing even more genes in its backbone, has been described in America and Europe in recent years [8,9].

Among the naturally owned or acquired virulent factors a bacteria can have, the formation of biofilms is of high interest from both a clinical and food safety point of view [10,11]. Biofilms are extracellular matrices formed by bacteria that help protect them from adverse environmental conditions or substances, like UV radiation, disinfectants, antibiotics, high pressure, or high salinity [12]. The first step in biofilm formation is the attachment of bacteria on biotic or abiotic surfaces. Then they start producing an extracellular matrix consisting of polysaccharides, structural proteins, cell debris, and nucleic acids, which are collectively referred to as extracellular polymeric substances (EPS). The structure is built so that there are nutrients, oxygen, and water flow within the biofilm, which sustain the bacterial populations in it and preserve their viability. Once the biofilm is settled, some inhabiting bacteria can exit the structure by freeing themselves into the environment, then they can remain as planktonic cells, unbound to any surface, or start new biofilm structures elsewhere [1]. Due to the isolating effect biofilms have on bacterial populations, antibiotic treatments can be hindered and lose effectiveness, worsening prospects for patients with bacterial infections encased in biofilms [13]. *Salmonella* species are known biofilm producers; the main concern regarding *Salmonella* biofilms is the diminishing impact they can have on cleaning and disinfection protocols throughout the food chain [14]. Thus, *Salmonella* Infantis is an emerging food-borne serovar with a critical implication on global health, highlighting the need for additional tools to control it.

The use of bacteriophages, viruses that naturally infect bacteria, is being studied as an antimicrobial strategy known as phage therapy. Their obligate nature requires them to infect bacterial hosts and destroy them to complete their replication lifecycle [15]. They are highly specific to hosts, meaning they are a tailored approach that, unlike antibiotics, only target the pathogen strain [16]. Their use is being implemented in numerous fields as therapeutic, prophylactic, and disinfecting agents, as an alternative to conventional antimicrobial substances [17,18,19]. Besides, bacteriophages can possess external enzymes capable of lysing biofilms called depolymerases; thus, their feature in newly isolated phages is deemed of interest for any phage therapy approach that could be challenged by a biofilm [20].

This study aims to provide insight into the individual and collective capacities three previously characterized bacteriophages possess to lyse biofilms formed by *S*. Infantis poultry field strains.

## 2. Results

### 2.1. Screening for Biofilm-Forming Salmonella Infantis—Colony Morphology on Congo Red Agar

**Figure 1:**
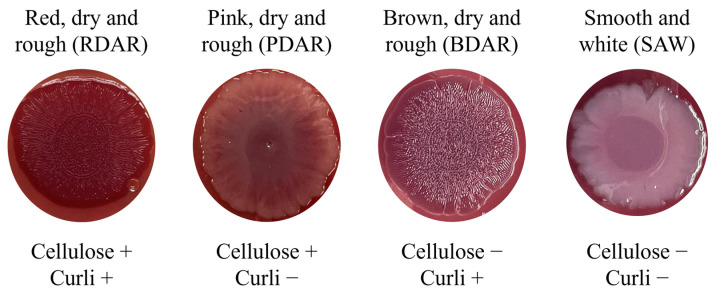
Colony morphology classification on Congo Red Agar. Each morphology is indicative of different EPS secretion patterns (cellulose and curli).

Captions of colony morphologies are shown in Figure 1. After the 96-h incubation at 25 °C on Congo Red agar (CRA) plates, 90.48% (19/21) of the field strains expressed morphologies consistent with EPS secretion (Table 1). Brown, Dry and Rough (BDAR) morphologies, indicative of curli secretion, were observed on 76.19% (16/21) of the field strains, Red, Dry and Rough (RDAR) morphologies, indicative of both curli and cellulose secretion, were observed on 9.52% (2/21) of the strains, and Pink, Dry and Rough (PDAR) morphologies, indicative of cellulose secretion, were found on 4.76% (1/21) of the strains. Smooth and White (SAW) morphologies, indicative of the absence of both cellulose and curli, were observed on 9.52% (2/21) of the field strains. The positive controls for biofilm production, ATCC14028 and NCTC12115, tested positive with RDAR and PDAR morphologies, respectively, while the negative control, ATCC23520, exhibited an SAW morphology.

However, after a 96-h incubation of the field strains at 37 °C on CRA plates, 85.71% (18/21) exhibited SAW morphologies, 14.28% (3/21) BDAR morphologies, and 4.76% (1/21) PDAR morphologies. ATCC14028 exhibited a PDAR morphology, while both NCTC12115 and ATCC23520 exhibited SAW morphologies.

### 2.2. Screening for Biofilm-Forming Salmonella Infantis—Quantification of Biofilm Production in 96-Well Microplates

**Figure 2:**
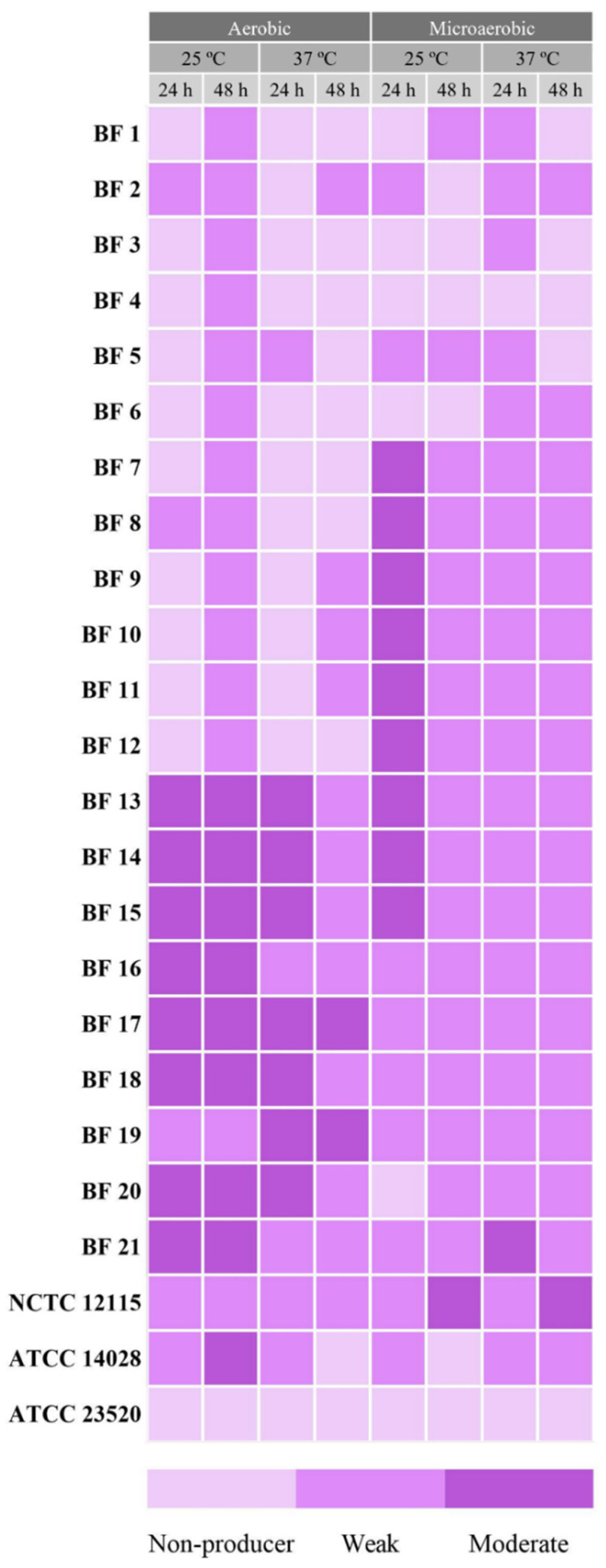
Biofilm-producing capacities of the *Salmonella* Infantis field strains. BF refers to *Salmonella* Infantis field strains and NCTC12115, ATCC14028, and ATCC23520 to reference strains used as controls.

The results of the biofilm quantification assays indicated that all field strains exhibited biofilm-producing capacities to varying degrees under at least one of the eight conditions (Figure 2). Overall, 71.43% (15/21 strains) showed moderate biofilm production, while 28.57% (6/21) exhibited weak biofilm production. Control strains ATCC14028 and NCTC12115 tested positive with moderate biofilm-producing capacities, and ATCC23520 tested negative for biofilm-producing capacities.

**Figure 3:**
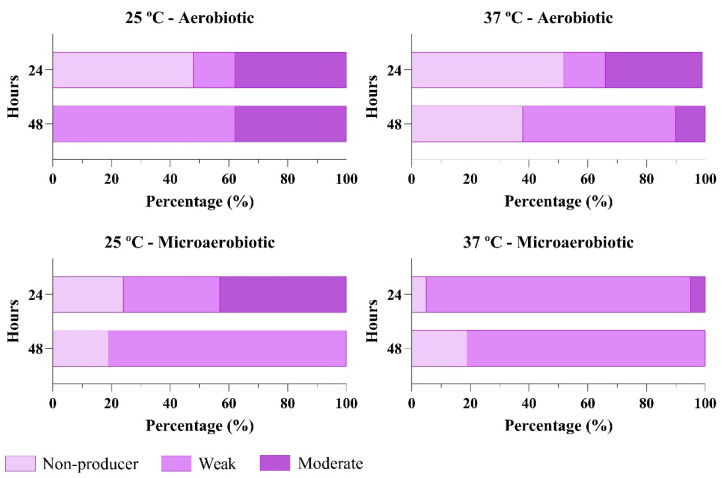
Percentages of biofilm-producing levels in each environmental condition. The graphs are grouped per environmental conditions, including 24 and 48 h.

Regarding the environmental and timing conditions, the highest percentage of biofilm formation was observed at 25 °C for 48 h under aerobic conditions, where 100% (21/21) of the field strains demonstrated biofilm-forming capacities (Figure 3). This was closely followed by 24 h at 37 °C under microaerobic conditions, with 95.24% (20/21) of the strains showing biofilm-forming capacities, and by both 37 °C and 25 °C for 48 h under microaerobic conditions, each yielding 80.95% (17/21) strains.

Regarding the degree of biofilm production, 42.86% (9/21) of the strains produced moderate biofilms at 25 °C for 24 h under microaerobic conditions, followed by 38.10% (8/21) at 25 °C for both 24 and 48 h under aerobic conditions.

### 2.3. Biofilm Characterization

Following the initial screening, strains BF 9 and BF 15 were selected for in-depth characterization of their biofilm-forming capacities. Biofilm formation was quantified in 96-well microplates using optical density (OD) measurements and determining *Salmonella* concentration via colony-forming units (CFU).

**Figure 4:**
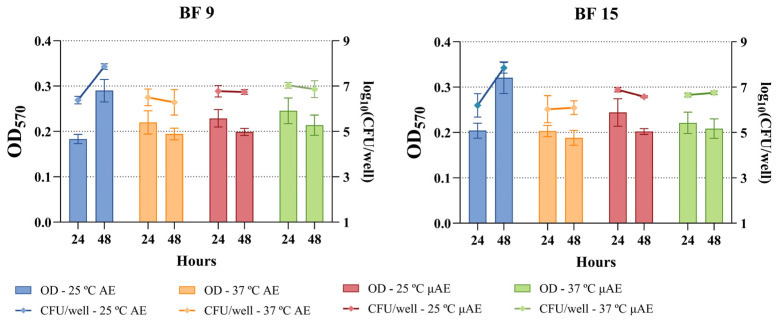
Characterization of the biofilm-producing abilities of BF 9 and BF 15. The graph shows the OD (570 nm) values and the CFU/well of the field strains under aerobic (AE) and microaerobic (µAE), at 25 and 37 °C after 24- and 48-h incubations. The left Y references the OD data and the right Y axis the CFU/well data.

Both strains exhibited relatively stable OD values and CFU counts after 24 and 48 h; however, a marked increase in both OD and CFU was observed after 48 h at 25 °C under microaerobic conditions (Figure 4).

### 2.4. Bacteriophage Selection and Propagation

In the bacteriophage selection assays to target BF 9 and BF 15, 4 out of 4 (100%) of the bacteriophages expressed lytic capacities on BF 9, but only 3 out of 4 (75.0%) had efficiency of plating values (EOP) above 1 (Table 2). As per BF 15, 3 out of 4 phages presented clear lysis on double layer agar (DLA) plates and presented EOP values above 1 (Table 2).

Bearing the results in mind, phages vB_Si_CECAV_FGS009, vB_Si_CECAV_FGS017, and vB_CECAV_044 were selected for the study and further propagated with their propagation strains. Afterwards, a 1:1:1 proportion cocktail (CKT) of the three candidates was created for the study.

### 2.5. Biofilm Disruption by Bacteriophages

From the 32 assays conducted to evaluate the bacteriophages’ capacities to target 48-h biofilms, the OD values of the phage-treated biofilms reflected significant decreases in 37.50% (12/32) of the assays and a significant increase in 3.13% (1/32) of the assays, when compared to the untreated controls (*p*-value < 0.05). Regarding *Salmonella* concentrations within phage-treated biofilms, 78.13% (25/32) of the assays showed a significant reduction (*p*-value < 0.05), and no significant increases (0/32) were observed in any assay (*p*-value > 0.05).

**Figure 5:**
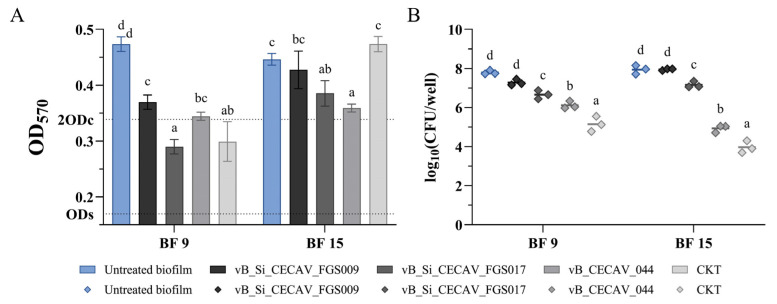
OD measurements (**A**) and *Salmonella* concentrations (**B**) of phage-treated biofilms at 25 °C in an aerobic environment. The graphs showcase untreated biofilms (in color) and treated biofilms (grayscale). abcd: superscripts indicate significant differences between groups (*p*-value ≤ 0.05). In (**A**), ODc and 2ODc are represented as dotted lines on the Y axis. Average values below ODc indicate no biofilm production, average values between ODc and 2ODc indicate weak biofilm production, and average values between 2ODc and 4ODc indicate moderate biofilm production. 4ODc = 0.678 (outside the Y axis range).

Under 25 °C in aerobic conditions, all BF 9 produced biofilms were reduced by the phage application (4/4), and, notably, the ones treated with vB_Si_CECAV_FGS017 and the CKT were reduced from moderate to weak. In BF 15 produced biofilms, only the ones treated with vB_Si_CECAV_FGS017 and vB_CECAV_044 (2/4) showed significant OD reductions (*p*-value < 0.05) (Figure 5A). In terms of *Salmonella* concentration, all treatments, except for phage vB_Si_CECAV_FGS009, resulted in statistically significant reductions (*p*-value < 0.05), with the most pronounced effect observed following treatment with the phage CKT (Figure 5B).

**Figure 6:**
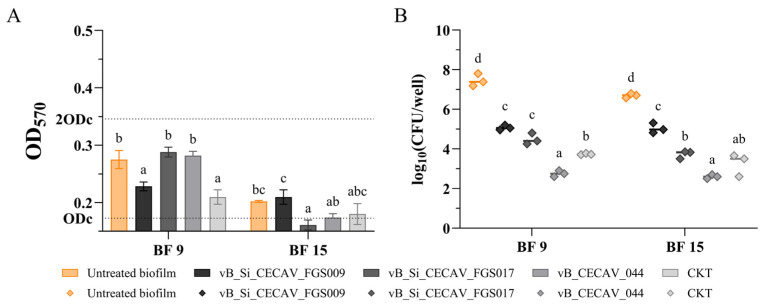
OD measurements (**A**) and *Salmonella* concentrations (**B**) of phage-treated biofilms at 37 °C in an aerobic environment. The graphs showcase untreated biofilms (in color) and treated biofilms (grayscale). abcd: superscripts indicate significant differences between groups (*p*-value ≤ 0.05). In (**A**), ODc and 2ODc are represented as dotted lines on the Y axis. Average values below ODc indicate no biofilm production, average values between ODc and 2ODc indicate weak biofilm production, and average values between 2ODc and 4ODc indicate moderate biofilm production. 4ODc = 0.691 (outside the Y axis range).

Biofilms produced at 37 °C under aerobic conditions showed the second-highest values for both OD and *Salmonella* concentrations (Figure 6). Notably, significant reductions were observed only in BF 9 biofilms treated with vB_Si_CECAV_FGS009 and the CKT, with 2 out of 4 assays showing a decrease. In contrast, significant OD reduction was observed in only 1 out of 4 assays for BF 15, specifically in those treated with vB_Si_CECAV_FGS017, where the biofilm level dropped from weak to none (Figure 6A). Regarding *Salmonella* concentration within the biofilms, all phage-treated groups presented significant reductions (4/4 for both strains) (*p*-value < 0.05), among them the ones treated with vB_CECAV_044 stood out with the highest reductions, up to 4 log units (Figure 6B).

**Figure 7:**
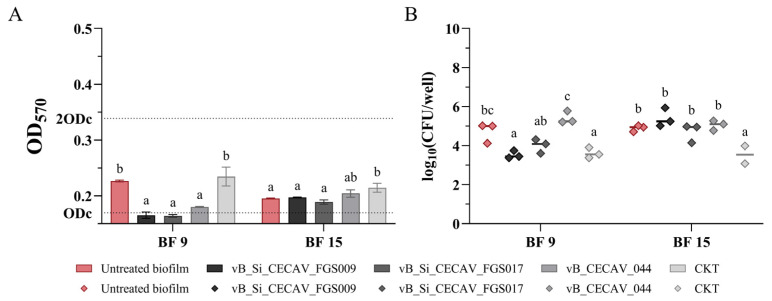
OD measurements (**A**) and *Salmonella* concentrations (**B**) of phage-treated biofilms at 25 °C in a microaerobic environment. The graphs showcase untreated biofilms (in color) and treated biofilms (grayscale). abc: superscripts indicate significant differences between groups (*p*-value ≤ 0.05). In (**A**), ODc and 2ODc are represented as dotted lines on the Y axis. Average values below ODc indicate no biofilm production, average values between ODc and 2ODc indicate weak biofilm production, and average values between 2ODc and 4ODc indicate moderate biofilm production. 4ODc = 0.678 (outside the Y axis range).

In the assays performed on the biofilms generated at 25 °C in microaerobic environments, 3 out of 4 phage-treated BF 9 biofilms exhibited significant reductions in OD values (*p*-value < 0.05), with the CKT-treated group being the only one without OD reductions (Figure 7A). Furthermore, in the groups where vB_Si_CECAV_FGS009 and vB_Si_CECAV_FGS017 were administered, the biofilms were completely reduced. From the phage-treated BF 15 biofilms OD values, 3/4 presented no significant reductions (*p*-value ≥ 0.05), and the group where the CKT was administered, OD values increased significantly (*p*-value < 0.05), although it was still categorized as weak. Regarding *Salmonella* concentration, vB_Si_CECAV_FGS009 and CKT were the only treatments that significantly reduced bacterial concentrations within biofilms from BF 9, while only the CKT-treated wells from BF 15 presented significantly reduced *Salmonella* concentrations (Figure 7B).

**Figure 8:**
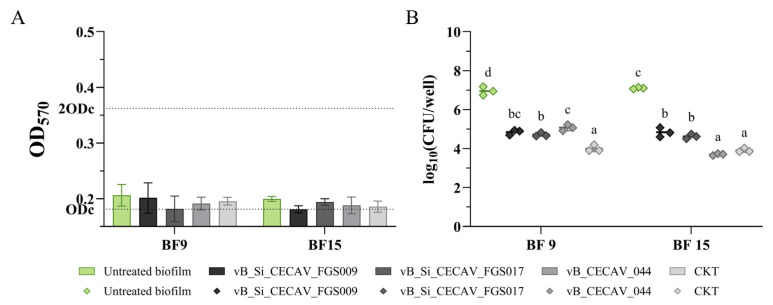
OD measurements (**A**) and *Salmonella* concentrations (**B**) of phage-treated biofilms at 37 °C in a microaerobic environment. The graphs showcase untreated biofilms (in color) and treated biofilms (black and white). abcd: superscripts indicate significant differences between groups (*p*-value ≤ 0.05). In (**A**), ODc and 2ODc are represented as dotted lines on the Y axis. Average values below ODc indicate no biofilm production, average values between ODc and 2ODc indicate weak biofilm production, and average values between 2ODc and 4ODc indicate moderate biofilm production. 4ODc = 0.725 (outside the Y axis range).

Regarding the phage-treated biofilms at 37 °C in microaerobic conditions, none presented differences in OD values that could indicate a significant decrease for both BF 9 and BF 15 (Figure 8A). Regarding *Salmonella* concentration, all the phage-treated groups were significantly reduced in both bacterial biofilms (Figure 8B).

Overall, assessing the number of tests where the OD values were reduced compared to the control group, vB_Si_CECAV_FGS017 reduced them in 50.0% of the assays (4/8), followed by vB_Si_CECAV_FGS009 and vB_CECAV_044, both with 37.5% (3/8), and by the CKT treatment, with 25.0% (2/8). On the other hand, the phage treatment with the highest reduction in incidence in *Salmonella* concentrations was the CKT treatment, with a 100% (8/8), followed by vB_Si_CECAV_FGS017 and vB_CECAV_044, both with a 75.0% incidence (6/8), and vB_Si_CECAV_FGS009 with a 37.5% incidence (3/8).

### 2.6. Biofilm Imaging with Scanning Electron Microscopy

**Figure 9:**
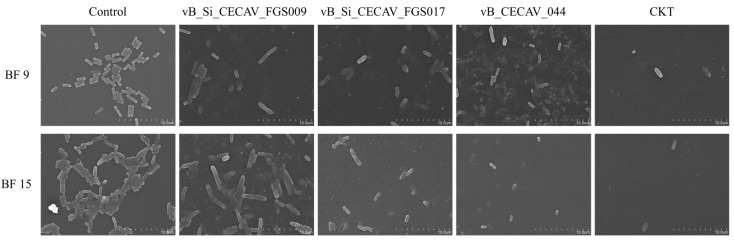
SEM captions of biofilms formed by BF 9 and BF 15 at 25 °C for 48 h. The figure shows untreated biofilm alongside those treated with each individual phage and the phage cocktail (CKT).

The Scanning Electron Microscopy (SEM) images revealed notable differences in cell density and morphology between phage-treated and untreated biofilms of BF 9 and BF 15 (Figure 9).

The cell size of *Salmonella* in untreated BF 9 biofilms was 1.47 ± 0.178 µm, but after treatment it increased significantly to 2.20 ± 0.44 µm and 2.40 ± 0.55 µm in biofilms treated with vB_Si_CECAV_FGS009, vB_Si_CECAV_FGS017, respectively (*p*-value < 0.05). No significant differences were observed with vB_CECAV_044 or the CKT treatment, 1.92 ± 0.60 µm and 2.12 ± 0.11 µm, respectively (*p*-value > 0.05). In BF 15 biofilms, the cell size was 1.73 ± 0.18 µm. Upon phage treatment, the cell size increased to 3.61 ± 0.57 µm in the vB_Si_CECAV_FGS009-treated biofilms (*p*-value < 0.05). No significant differences in size were found with vB_Si_CECAV_FGS017-treated biofilms (1.82 ± 0.22 µm), vB_CECAV_044 (1.10 ± 0.22 µm), or CKT (1.89 ± 0.11 µm) (*p*-value > 0.05).

## 3. Discussion

*Salmonella* Infantis is becoming an increasingly difficult serovar to eliminate, partly due to its ability to persist in facilities even after routine cleaning and disinfection procedures [8]. Contaminated materials such as drinkers, feeders, or any surfaces that are difficult to clean and disinfect can act as persistent sources of contamination if not properly sanitized between production cycles [21]. The ability to produce biofilms from *Salmonella* strains only increases the likelihood of persistence in the facilities, thus increasing the risk of transmission despite the regular cleaning and disinfection protocols between batches of animals [22]. Rather than the ability or inability to produce biofilms, several studies have linked the capacity for biofilm production to growth conditions such as incubation temperature, nutrition, and osmolarity [23]. Therefore, given the relevance of these factors in animal rearing and food processing, this study evaluates the biofilm-producing capacities of *S*. Infantis field strains under 25 and 37 °C.

The results from the CRA plates at 25 °C indicated that 90.48% of the strains expressed EPS secretion, while only 19.04% expressed so at 37 °C (14.28% BDAR and 4.76% PDAR). At 25 °C, 4.76% of the field strains in this study expressed PDAR morphologies and 9.52% RDAR morphologies, both of which indicate cellulose production. As an EPS component, cellulose has been described as a major element in the structural organization of the biofilms that can contribute to bacterial resistance, enhancing bacterial survival against commonly used disinfectants [23,24,25]. In addition, the strains with RDAR morphologies, together with 76.19% of the strains that expressed BDAR morphologies, indicate the production of curli, a key element in the first stages of biofilm formation, providing permanent adhesion to abiotic surfaces [26]. The results from this study align with previous reports, such as Vivek et al., 2016, who described a 91.03% incidence of EPS expression on CRA plates at 22 °C, and 100% absence of EPS expression at 37 °C among 145 *Salmonella* field strains from different serovars [27]. These findings highlight the strong influence of temperature on EPS secretion and biofilm-associated morphology expression in *Salmonella* Infantis, underscoring their adaptive strategies to survive in different environmental conditions.

Moreover, despite none of the strains being strong biofilm producers in the 96-well plate assays, all of them exhibited biofilm-producing abilities under one of the four tested environmental conditions. Although further studies should be carried out to affirm so, the widespread presence of the pESI and pESI-like plasmids within the serovar Infantis could be the cause of this generalized biofilm-producing ability, since among the virulence factors it carries, enhanced biofilm production is one of them [8,9,28]. The assays performed at 25 °C for 48 h under aerobic conditions were the ones with the highest incidence of biofilm production, which also aligns with what is described of *Salmonella*’s biofilm-producing abilities in the literature [28]. While biofilm production was observed under microaerobic conditions, the in-depth characterization of the BF 9 and BF 15 biofilms revealed that those were weak, near the non-producing limit (ODc). Since broiler rearing facilities operate at temperatures above 30 °C or above during the first part of the production cycles, less biofilm-encased *Salmonella* strains might be expected on surfaces during that period. However, as the cycle progresses, temperatures are gradually adjusted to 20 °C to adapt to the animals’ growth and ensure thermal comfort [29]. According to the results of this study, these cooler settings may promote biofilm formation and lead to stronger biofilm patterns. Furthermore, increased biofilm production could occur between production cycles, when facilities are unoccupied and maintained at room temperature for cleaning and disinfecting protocols.

The efficacy of bacteriophages as surface disinfectants has been demonstrated in different stages of the food-chain [30,31]. Sevilla-Navarro, 2024 demonstrated that two bacteriophage applications in poultry houses, spaced 24 h apart, achieved complete *Salmonella* reductions when *Salmonella* was below 5 log_10_ (CFU/sample) but only achieved partial reductions when it was above 5 log_10_ (CFU/sample) [32]. The results from the current study could offer a comprehensive response to the incomplete elimination of the bacteria in those facilities after the two-times phage application. The CFU/well assessment of BF 9 and BF 15 biofilms in the biofilm characterization showed that, despite borderline negative OD results that would indicate weak biofilm production, *Salmonella* was recovered at concentrations of up to 5 log10 (CFU/well). This underscores the relevance of biofilms as reservoirs of resistant *Salmonella* in the environment.

In the biofilm disruption assays, bacteriophages reduced biofilm biomass in 37.50% of the cases. While the phage vB_Si_CECAV_FGS017 showed the highest incidence of biofilm reduction, all tested candidates demonstrated the ability to disrupt biofilms to some extent. Their effectiveness as biofilm-disrupting agents is supported by previous characterization, which identified depolymerase enzymes in the structure of all phages [32,33]. Notably, in some biofilm disruption assays, the CKT-treated wells presented minimal to no reduction of biofilms compared to the wells treated with individual phages. For instance, at 25 °C under aerobic conditions, BF 15 biofilms were efficiently reduced by vb_CECAV_FGS017 and vb_CECAV_044, while vb_CECAV_FGS009 and CKT treatments did not result in biofilm reduction. In this study, phages were individually selected by their host range and lytic capacities against BF 9 and BF 15, and then CKT was assembled with equal parts of each phage. While the design of bacteriophage cocktails is extensively described in the literature to attain optimized phage performances, these are usually based on the lytic capacities of the phages, and not on their potential towards disrupting biofilm matrices [34,35]. In that sense, coupled with phage cocktail design tools, which aim to avoid antagonistic or competitive relationships between phages, genomic analysis of the phages and bacterial strain may benefit the outcome of the phage application. Specifically, understanding depolymerase production of the phages and whether they target EPS compounds of the biofilm-producing strains would provide valuable insights to the results of this study [36]. However, the phages were able to penetrate the biofilm matrix and infect the embedded *Salmonella* cells. Among the phages tested, CKT achieved the highest performance, with significant reductions in *Salmonella* concentrations in 100% of the assays. An indicative sign that corroborates *Salmonella* reduction by phage lysis is the increase in cell size observed through SEM imaging. Since some phages, like ZCSE2 or λ, can hinder or hijack the host’s metabolic mechanisms, an increase in size on phage-infected cells can occur prior to bacterial lysis, primarily due to inhibition of division in matured cells [37,38].

However, some assays showed results consistent with potential resistance mechanisms, possibly mediated by quorum sensing [39,40,41]. These cases were characterized by minor reductions in CFU counts but marked decreases in OD values—suggesting reduced bactericidal activity, while depolymerase-mediated biofilm degradation remained unaffected. Moreover, the phage candidates expressed changing lytic patterns depending on the environmental conditions. For instance, under aerobic conditions, phage vB_Si_CECAV_FGS009 effectively reduced *Salmonella* concentration in BF 9 biofilms grown at 37 °C, but lost its efficacy at 25 °C. Such changing patterns also occurred under the same environmental conditions, like under microaerobic conditions, where phage vB_Si_CECAV_FGS009 was able to reduce biofilms (OD) and *Salmonella* concentrations of BF 9 biofilms, but lost its efficacy to do both in BF 15 biofilms. As membrane receptors can vary in abundance depending on the physiological needs of the host, and these are also modulated by environmental factors, like temperature or atmospheric conditions, phage efficacy can depend directly on environmental conditions, since phages target specific membrane receptors [42]. Hence, the results of this study highlight the critical importance of considering environmental and incubation conditions when evaluating the efficacy of bacteriophage treatments against *Salmonella* Infantis biofilms. The changing efficacy patterns suggest that evaluating phage candidates solely under ideal laboratory conditions could lead to overestimating their efficacy, especially in the context of biofilm-associated infections. While laboratory conditions (37 °C) facilitate phage growth and standardization, they may not reflect the actual environmental settings found in production facilities, where lower temperatures and limited oxygen availability, like cracks, water lines, or poorly ventilated areas, are common.

Overall, the three bacteriophages proved to have both lytic capacities towards biofilm-embedded *Salmonella* Infantis strains and biofilm-disrupting capacities that can reduce EPS structures that protect the bacteria. However, the results also highlight that phage selection should not be done solely on host range and lytic activity, but also their performance under environmental conditions that mimic those encountered in farm settings. Furthermore, other stages of the food chain with continuous and intense workflows, like slaughterhouses or hatcheries, could benefit from the integration of phages in their cleaning and disinfection protocols to control bacterial populations and limit pathogen transmission.

## 4. Materials and Methods

### 4.1. Bacterial Collection

A total of 21 *Salmonella* Infantis field strains were recovered from CECAV’s repository (Centro de Calidad Avícola y Alimentación Animal de la Comunidad Valenciana, Spain). All strains were isolated from boot swabs collected at broiler farms as part of the self-control samples within Spain’s national *Salmonella* control programs between 2020 and 2023. The strains preserved in a 20% glycerol suspension were streaked in Nutritive agar (VWR-Avantor, Radnor, PA, USA) and incubated overnight at 37 °C before use.

### 4.2. Environmental Conditions

Aiming to assess the feasibility of bacteriophages against *Salmonella* Infantis under different field conditions, four different environments were emulated throughout the study. The assays focused on the capacities of *Salmonella* Infantis strains to form biofilms at 25 and 37 °C, also assessing their capacities under aerobic and microaerobic environments. Through the mixture of these four environmental variables, four conditions were established: (A) 25 °C with aerobic atmosphere, (B) 37 °C with aerobic conditions, (C) 25 °C with microaerobic conditions, and (D) 37 °C with microaerobic conditions.

Aerobic conditions were attained through conventional incubations with unmodified atmospheres, while microaerobic conditions were attained through incubations in hermetic containers with CampyGenTM 3.5 L sachets (ThermoScientific, Waltham, MA, USA), which generate atmospheres with 8–9% O_2_ and 7–8% CO_2_.

### 4.3. Screening for Biofilm-Forming Salmonella Infantis—Morphology by Congo Red Agar Assay

The colony morphology of the *Salmonella* Infantis field strains was evaluated on CRA for curli, fimbriae, and cellulose production, as described by Vivek et al., 2016, with some modifications [27]. In brief, overnight cultures of the field strains were prepared on 5 mL of LB broth (without NaCl) (Sharlab, Sentmenat, Spain). After incubation, 10 µL were laid on the surface of CRA plates (LB broth without NaCl, 15% agar powder (VWR-Avantor, Radnor, PA, USA), 0.4 µg/mL Congo Red (ThermoScientific, MA, USA), and 0.2 µg/mL Brilliant Blue R (ThermoScientific, Waltham, MA, USA). Additionally, ATCC14028 and NCTC12115 (both *Salmonella enterica* subsp. *enterica* serovar Typhimurium) were used as positive biofilm-producing strain controls, and ATCC23520 (*Escherichia coli*; serovar O28a,28c:K73 (B18):H-) as a negative control. The inoculated plates were incubated at 25 and 37 °C for 96 h under aerobic conditions, and the assay was performed in triplicate.

After the 96-h inoculation, colony morphologies were categorized according to their appearance: RDAR, PDAR, BDAR, SAW. The morphological classification of the colonies was indicative of two major EPS substances, cellulose and curli [23].

### 4.4. Screening for Biofilm-Forming Salmonella Infantis—Quantification of Biofilm Production in 96-Well Microplates

To quantify the biofilm-producing capacities of the 21 *Salmonella* Infantis field strains, a protocol previously described by Korzeniowski et al., 2022, was followed with slight modifications [21]. To that aim, 5 mL inoculums of the field strains were prepared on LB broth (without NaCl), and after an overnight incubation, the inoculums were adjusted to 0.2 (10^8^ CFU/mL) OD at 600 nm. Then 200 µL of each inoculum was transferred to a 96-well microplate in triplicate and incubated under the four established environmental conditions for 24 and 48 h. After incubation, the contents of the wells were discarded and the wells were washed with 250 µL of 1× Phosphate Buffered Saline (PBS, TH.GEYER, Renningen, Germany) (0.14 M NaCl, 0.0027 M KCl, and 0.01 M PO4^−3^, pH 7.4) three times to rinse off any planktonic cells. Then, 200 µL of methanol (Chem-lab, Zedelgem, Belgium) was added to each well, to fixate the biofilms to the well walls, and incubated for 15 min at room temperature. After the methanol was removed, 200 µL of 0.5% (*w*/*v*) crystal violet (BioGnost, Zagreb, Croatia) was added to stain the biofilm structures in the wells. After a 5 min incubation at room temperature, the wells were emptied and washed twice with sterile distilled water. Lastly, 200 µL of ethanol 96% (*v*/*v*) (Chem-lab, Zedelgem, Belgium) was added to the wells and left for 10 min to dissolve the stained biofilms. The OD of the wells was individually measured at 570 nm using a Multiskan Skyhigh Microplate reader (ThermoScientific, Waltham, MA, USA). Additional wells filled with 200 µL of LB broth (without NaCl), free from *Salmonella*, were also added and processed as described above to be used as negative control wells.

The OD values obtained from the control wells were used to calculate the control OD (ODc) values with the following formula: ODc = (average of control OD wells) + 3 (standard deviation of control OD wells). Then an OD for *Salmonella* inoculated wells was obtained by calculating the average of the OD (ODs). The biofilm-forming capacities of the strains were then classified depending on the following brackets: non-producer (ODs ≤ ODc), weak (ODc < ODs ≤ 2ODc), moderate (2ODc < ODs ≤ 4ODc), and strong (4ODs < ODc).

ATCC14028 and NCTC12115 (both *Salmonella enterica* subsp. *enterica* serovar Typhimurium) were used as positive biofilm-producing strain controls, and ATCC23520 (*Escherichia coli*; serovar O28a,28c:K73 (B18):H-) as a negative control.

### 4.5. Characterization of Biofilm

After the screening for biofilm production in the *Salmonella* Infantis amid the field strains, BF 9 and BF 15 were selected to proceed with further studies and characterized. Their biofilm-producing capacities were characterized under the four previously stated environmental conditions, again for 24 and 48 h. To do so, their biofilm productions were quantified using the 96-well plate assays previously described, and the CFU/well were assessed as described by Sevilla-Navarro et al. [43]. In brief, after incubation, the loaded wells were washed with 1× PBS three consecutive times, and the contents of each well were individually scraped and resuspended in 250 µL of PBS [44]. The suspended biofilms here were then thoroughly vortexed, and serial dilutions were performed to assess *Salmonella* concentration on the surface of Xylose–Lysine–Desoxycholate agar (XLD) (Sharlab, Sentmenat, Spain).

### 4.6. Bacteriophage Selection and Propagation

A total of 4 previously characterized bacteriophages from CECAV’s repository were tested through the DLA method to establish their EOP on the selected field strains, BF9 and BF 15 (Table 3) [32,33].

To do so, 200 µL of bacterial inoculums at OD 0.2 (600 nm) of the propagation strains and the field strains were mixed with 10 µL of phage dilutions and incubated at room temperature for 15 min. Then 5 mL of LB broth (acc. Miller) (Sharlab, Sentmenat, Spain) supplemented with agar powder at 0.6% was added to the incubated aliquots and poured on top of LB agar (acc. Miller) plates (Sharlab, Sentmenat, Spain). After overnight incubations at 37 °C, PFU/mL titers were assessed, and the EOP of the phages with the challenge strains was determined relative to the titer attained with their propagation strain. The phages that exhibited clear plaques and expressed EOP values ≥ 1 with the challenge strains’ bacterial lawns were selected for the study.

Phage preparation of the selected phages was performed with their propagation strains as described by Torres-Boncompte et al., 2025, [33]. In brief, a 5 mL inoculum of the propagation strains at 0.2 OD (600 nm) was added to 45 mL of fresh LB broth (acc. Miller) with a phage aliquot to reach a multiplicity of infection of 0.01. After a 4-h incubation, phages were harvested, centrifuged (8,000× *g* for 15 min at 4 °C) and filtered through 0.45 and 0.22 µm PES filters. Then phage titers were assessed through the DLA method, and the phage aliquots were stocked at 4 °C until use.

### 4.7. Biofilm Disruption by Bacteriophages

To assess the biofilm-disrupting capacities of the selected phages, biofilms from BF 9 and BF 15 were generated under the same four different environmental conditions, as previously described. Afterwards, the procedure described by Hosny et al. (2023) was followed with slight modifications to test the three phage-candidates and the CKT [45]. After the 48-h incubations, the wells were washed with 1× PBS three times to wash off any planktonic cells, then 200 µL of phage aliquots were added to reach 10^8^ PFU/well. Plates incubated at 25 °C were then incubated for an additional 8 h, while the ones incubated at 37 °C were incubated for 4 more hours. After the respective incubations, the wells were washed with 1× PBS three times, and the remaining biofilms were scraped off the walls and bottom of the wells and resuspended in 250 µL of 1× PBS. Then, *Salmonella* concentration on the samples was assessed as previously described in XLD plates. Crystal violet assays were simultaneously performed to quantify the biofilms on the wells, following the same methodology previously stated.

### 4.8. Biofilm Imaging with Scanning Electron Microscopy

Bearing the results from the biofilm disruption by the bacteriophages assay, biofilms from BF 9 and BF 15 formed at 24 °C after 48 h were processed for SEM imaging. To do so, the protocols described by Thames et al., 2023, were followed with slight modifications [46]. First, cultures of BF 9 and BF 15 were incubated overnight at 37 °C under aerobic conditions in LB broth (without NaCl). After incubation, the inoculums were adjusted to OD 0.2 (10^8^ CFU/mL) at 600 nm, and 2 mL of each was transferred to wells from 24-well plates. Then, polystyrene (PS) coupons of 1 × 1 cm, previously sterilized at 120 °C for 15 min, were individually added to the wells. Additionally, a portion of the coupon-loaded wells were filled with 2 mL of sterile LB broth (without NaCl) to form a negative control group. After the 48-h incubation at 25 °C, the biofilms were divided into two groups: a control positive group, with untreated biofilms, and treatment groups, where phages were administered. The biofilm-coated PS coupons from the treatment groups were first rinsed with 1× PBS to eliminate planktonic cells and transferred to sterile wells, where 2 mL of the selected phages and the CKT were administered at 10^8^ PFU/well.

After an 8-h incubation, at 25 °C under aerobic conditions, all coupons were subtracted from the wells, rinsed with 1× PBS, and fixed in a 2.5% (*v*/*v*) glutaraldehyde for 60 min. Then, the samples were dehydrated using an ethanol gradient (10, 30, 50, 75, and 95%, 10 min each) [47]. The coupons were coated with Pd/Au and observed in a Hitachi S4800 electron microscope (SCSIE, Universitat de València, Valencia, Spain).

The cell size measurements from the SEM images were taken with the ImageJ 1.54p software [48].

### 4.9. Statistical Analysis

The statistical analysis performed for the biofilm disruption by bacteriophages was carried out with 2-way ANOVA models (multiple comparisons), both for the OD values and the CFU/well values. Furthermore, all CFU/well values were converted to log10 (CFU/well) for data analysis. The cell size measurements from the SEM images were also analyzed with 2-way ANOVA models (multiple comparisons). A significance level of *p*-value ≤ 0.05 was adopted to denote statistical significance in all analyses. All statistical analyses were performed using GraphPad Prism 10.

## 5. Conclusions

The bacteriophages of the present study proved to be promising candidates for enhanced phage therapy application aimed at disinfecting abiotic surfaces. Their lytic capacities towards *Salmonella* Infantis, coupled with their disrupting capacities towards biofilms, establish them as valuable antimicrobial agents that could mitigate the burden of *Salmonella* Infantis in the poultry sector and throughout downstream stages of the food chain. The study also emphasizes that successful phage therapy in animal production must account for the application context. Environmental variables such as temperature, oxygen levels, and surface type should be integrated into phage screening protocols to ensure that selected phages maintain their efficacy under the specific challenges posed by real production systems.

## Figures and Tables

**Table 1:** Colony morphology on CRA plates of the *Salmonella* Infantis isolates at 25 and 37 °C. RDAR: red, dry, and rough (cellulose+, curli+). PDAR: pink, dry and rough (cellulose+, curli−). BDAR: brown, dry, and rough (cellulose−, curli+). SAW; smooth and white (cellulose−, curli−).

Strain		25 °C	37 °C
*Salmonella* Infantis field strains (N = 21)	BF 1	BDAR	SAW
	BF 2	BDAR	SAW
	BF 3	BDAR	SAW
	BF 4	RDAR	PDAR
	BF 5	RDAR	SAW
	BF 6	BDAR	SAW
	BF 7	BDAR	SAW
	BF 8	BDAR	SAW
	BF 9	BDAR	BDAR
	BF 10	BDAR	SAW
	BF 11	BDAR	SAW
	BF 12	BDAR	SAW
	BF 13	BDAR	BDAR
	BF 14	BDAR	SAW
	BF 15	BDAR	BDAR
	BF 16	BDAR	SAW
	BF 17	BDAR	SAW
	BF 18	BDAR	SAW
	BF 19	SAW	SAW
	BF 20	PDAR	SAW
	BF 21	SAW	SAW
Control strains (N = 3)	ATCC14028	RDAR	PDAR
	NCTC12115	PDAR	SAW
	ATCC23520	SAW	SAW

**Table 2:** Bacteriophages from CECAV’s repository tested for lytic activity against the challenge strains BF 9 and BF 15. Positive results are expressed as “+”, and negative results as “−”. EOP: efficiency of plating.

Bacteriophage	BF 09			BF 015		
	Clear Lysis	Depolymerase Expression	EOP	Clear Lysis	Depolymerase Expression	EOP
vB_Si_CECAV_FGS009	+	+	1.03	+	+	1.03
vB_Si_CECAV_FGS017	+	−	1.46	+	−	1.32
vB_CECAV_041	+	−	0.72	−	−	-
vB_CECAV_044	+	+	1.03	+	+	1.00

**Table 3:** Bacteriophages from CECAV’s repository tested for lytic activity against the challenge strains BF 9 and BF 15.

Bacteriophage	Accession Number	Host Strain	Reference
vB_Si_CECAV_FGS009	PP407513	*S.* Infantis	[32]
vB_Si_CECAV_FGS017	PP429239	*S.* Infantis	
vB_CECAV_041	PQ064465	*S.* Kedougou	[33]
vB_CECAV_044	PQ220373	*S.* Infantis	

## Data Availability

Data will be made available on request.
